# Optimizing vaccine uptake in sub-Saharan Africa: a collaborative COVID-19 vaccination campaign in Madagascar using an adaptive approach

**DOI:** 10.1186/s13012-024-01412-5

**Published:** 2025-01-09

**Authors:** Viola Pavoncello, Irina Kislaya, Diavolana Koecher Andrianarimanana, Valentina Marchese, Rivo Rakotomalala, Tahinamandranto Rasamoelina, Simon Veilleux, Ariane Guth, Alexina Olivasoa Tsiky Zafinimampera, Sonya Ratefiarisoa, Olivette Totofotsy, Cheick Oumar Doumbia, Rivomalala Rakotonavalona, Holinirina Ramananjanahary, Zely Arivelo Randriamanantany, Jürgen May, Rivo Andry Rakotoarivelo, Dewi Ismajani Puradiredja, Daniela Fusco

**Affiliations:** 1 Research group: Implementation Research, Bernhard Nocht Institute for Tropical Medicine, Hamburg, Germany; 2 German Centre for Infection Research (DZIF), Hamburg-Borstel-Lübeck-Riems, Germany; 3 Department of Infectious Diseases Epidemiology, Bernhard Nocht Institute for Tropical Medicine, Bernhard-Nocht-Strasse 74, 20359 Hamburg, Germany; 4 Centre Hospitalier Universitaire (CHU) Androva, Mahajanga, Madagascar; 5 Centre d’ Infectiologie Charles Mérieux (CICM), University of Antananarivo, 101 Antananarivo, Madagascar; 6 University Clinical Research Center, University of Sciences, Techniques and Technologies of Bamako, Bamako, Mali; 7 Vaccination Program, Ministry of Public Health, Antananarivo, Madagascar; 8 Unit for the Coordination of the Covid-19 Vaccination, Ministry of Public Health, Antananarivo, Madagascar; 9 Ministry of Public Health, Antananarivo, Madagascar; 10 Department of Infectious Diseases, University of Fianarantsoa Andrainjato, 301 Fianarantsoa, Madagascar

**Keywords:** Dynamic Sustainability Framework, Vaccination campaigns, Vaccine uptake, Capacity strengthening, Resource-limited settings, COVID-19

## Abstract

**Background:**

The COVID-19 pandemic has highlighted the need for more effective immunization programs, including in limited resource settings. This paper presents outcomes and lessons learnt from a COVID-19 vaccination campaign (VC), which used a tailored adaptive strategy to optimise vaccine uptake in the Boeny region of Madagascar.

**Methods:**

Guided by the Dynamic Sustainability Framework (DSF), the VC implementation was regularly reviewed through multi-sectoral stakeholder feedback, key informant interviews, problem-solving meetings, and weekly monitoring of outcome indicators to identify and apply key adaptations. Qualitative data on processes were collected and analysed using a rapid assessment approach. Outcome indicators, including pre- and post-VC vaccine hesitancy and trends in vaccine doses administered, were analysed using generalized linear models. Additionally, vaccination coverage, geographic reach, and target population characteristics, and sustainability indicators, such as staff trained, facilities equipped, and degree of integration of operational and educational materials were also tracked.

**Results:**

Key strategy adaptations included using a proactive campaign approach, community-led awareness and outreach, particularly in remote areas, and addressing cold chain, waste management, vaccine transport, and information technology (IT) equipment gaps. Over six months, 24,888 COVID-19 vaccines were administered. The adapted strategy led to an 8% increase in doses administered weekly (RR = 1.08, CI 95%: 1.01-1.15). However, vaccine hesitancy among the unvaccinated population remained unchanged (∆ = 0.02, CI 95%: -0.04-0.08). In terms of sustainability, 340 staff were trained, and 10 primary healthcare facilities were equipped and refurbished.

**Conclusions:**

Implementing collaborative, multi-sectoral vaccination strategies that integrate healthcare services with proactive outreach and community-driven campaigns are effective in increasing vaccine coverage in resource-limited settings. It demonstrates how theory-based adaptive strategies can enhance vaccination rates, even if they do not significantly impact COVID-19 vaccine hesitancy within the community. More generally, this initiative has important implications for adult vaccination programmes other than those related to COVID-19.

**Supplementary Information:**

The online version contains supplementary material available at 10.1186/s13012-024-01412-5.

Contributions to the literature

- Despite a growing recognition that adaptation of health interventions to the context and continuous optimization through learning and evaluation are necessary to maximize their effectiveness, more guidance is needed on how to put this dynamic process into practice.
- This study provides an example of how the Dynamic Sustainability Framework can be leveraged to guide the implementation of a COVID-19 vaccination campaign in resource-limited settings.
- Our findings suggest that real-time evaluation and key adaptations of the initial strategy in response to contextual changes can be effective and that multisectoral stakeholder engagement and fostering local ownership are crucial for the success and sustainability of implementation.

## Background

The launch of global COVID-19 vaccination campaigns had generated hope that the necessary 70% global vaccination coverage threshold as set by the World Health Organization (WHO) could be achieved by June 2022 [1]. However, COVID-19 vaccines that are safe and effective can only be meaningful to the global community if they are accessible in an equitable and timely manner. Despite the extraordinary achievement of delivering vaccine candidates to the wider market within a short time span of one-year, multiple challenges, particularly for low- and middle-income countries (LMICs), led to disparities in COVID-19 vaccine access and uptake.

Regardless of international initiatives to structure the development and equitable distribution of vaccines, channeled through the COVID-19 Vaccines Global Access (COVAX) initiative, initially, serious supply shortages and national procurement methods of some countries that bypassed COVAX hindered the optimal function of the initiative in delivering timely and adequate doses to participating countries [2]. When the pace of COVID-19 vaccine production picked up at the beginning of 2022 and larger volumes of vaccine became available, public health systems, especially also in settings with limited resources, were not always prepared in terms of the logistics and vaccine supply management needed [3]. In addition, ensuring widespread COVID-19 vaccine acceptance is just as important as the strive towards equitable vaccine access. Further, vaccine hesitancy can be a significant barrier to achieving sufficient COVID-19 immunization coverage and was reported to be high in a number of countries, including many in sub-Saharan Africa (SSA) [4, 5].

In Madagascar, as of 23 November 2023, a total of 2,710,365 individuals received at least one dose of COVID-19 vaccine, reaching 10% of population coverage, one of the lowest COVID-19 vaccination coverage rates worldwide [6]. After a delay in joining the COVAX initiatives [3] on April 3, 2021, Madagascar initiated the necessary steps, and first doses were distributed on May 10, 2021. Complex political and policy developments [7], limited infrastructural capacity, as well as financial and operational challenges [8, 9] were some of the main reasons for Madagascar’s low COVID-19 vaccination coverage [10, 11]. While the introduction of targeted initiatives [7, 8] to reinforce governmental interventions and strengthen capacity helped boosting the vaccination rate in Madagascar to some extent, they remained isolated efforts, which were not sufficiently harmonized to achieve critical mass and guarantee a continuity in vaccine administration. In order to further increase COVID-19 vaccine coverage in Madagascar, several international initiatives were implemented, including the ‘COVID-19 vaccination campaign in the Boeny region of Madagascar: paving the road for worldwide vaccination coverage goal’ (CoBoGo) [12].

The CoBoGo vaccination campaign was a collaborative initiative by the Malagasy Ministry of Health (MoH) in partnership with Malagasy academic institutions and the Bernhard Nocht Institute for Tropical Medicine (BNITM) under the financing umbrella of the Deutsche Gesellschaft für Internationale Zusammenarbeit GmbH, which aimed to increase vaccine coverage in the Boeny region of Madagascar by 2.5% within six months. The campaign took advantage of the expertise built over the years among the Malagasy academia and the BNITM in the implementation of operational research projects in the region [13].

Recognizing that the implementation of such campaigns in a sustainable manner is a dynamic process, which needs to be responsive to complex and changing real-world healthcare settings and systems, the campaign was implemented according to an adaptive approach based on the principles of the Dynamic Sustainability Framework (DSF) [14, 15]. The DSF argues for the continuous refinement and improvement of interventions, through learning and evaluation, problem solving and ongoing adaptations to enhance fit between interventions, practice contexts and ecological systems over time [14]. However, there is limited guidance available on how to put this dynamic process into practice, especially in terms of incorporating necessary changes to the initial implementation strategies [16].

With this study, we aim to address this gap by describing how the dynamic process of our implementation strategy was operationalized, and identify key adaptations that can facilitate sustainability, and on-going optimization and improvements, in terms of COVID-19 and other adult vaccinations coverage and uptake. These findings can provide guidance for future mass vaccination campaigns in the region, and similar limited resource settings.

## Methods

### Conceptual framework

**Fig. 1:**
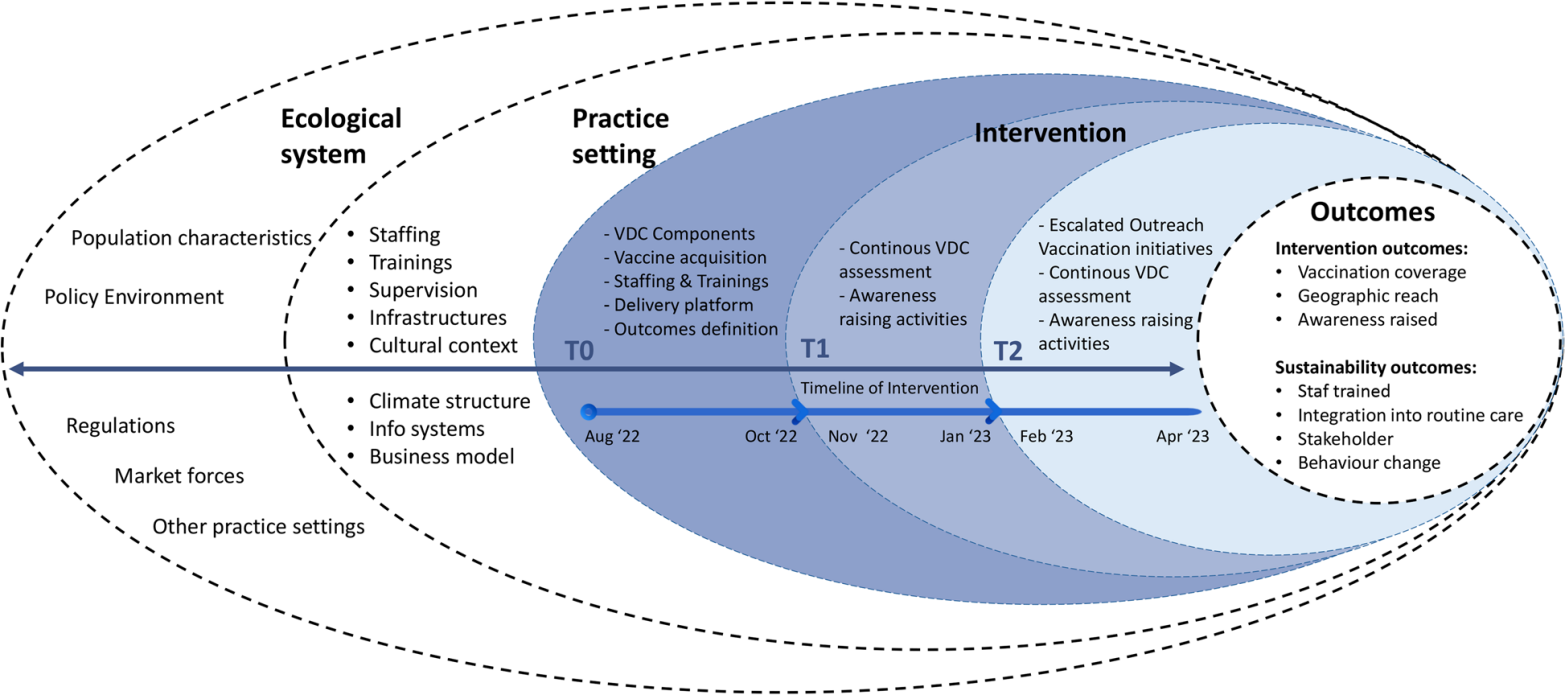
The dynamic sustainability framework adapted to the CoBoGo implementation campaign

The CoBoGo campaign adapted the DSF by Chambers et al. [14]. to conceptualise the domains that influence its implementation process and outcomes (Fig. 1). The theoretical framework provides a dynamic approach to understanding the evolution of interventions over time. It locates the intervention and its outcomes (Fig. 1, right part) within two ideal domains: the (i) practice setting/context of the intervention, and (ii) the ecological system (Fig. 1, left part). These domains indicate that different types of factors may be influential at different levels over the course of an intervention, although the directionality of influence is not conceived as linear or irreversible. This requires that implementation strategies are adapted over time (T0, T1, T2) to ensure the best fit between the vaccination campaign and the implementation setting.

### The intervention: overview of the campaign

The CoBoGo vaccination initiative was implemented in the Boeny region in the North-West of Madagascar and consisted of three phases: 1) a pre-campaign phase from August to October 2022 which involved vaccine acquisition, an initial vaccination distribution chain (VDC) needs assessment, as well as staff selection and training and evaluation of population attitudes regarding COVID-19 vaccination; 2) vaccination campaign Phase 1 using a passive approach in combination with an awareness raising campaign between October 31st, 2022 and February 15th, 2023, and 3) vaccination campaign Phase 2, which continued with VDC needs assessment and awareness raising activities but adopted a more proactive approach involving increased outreach initiatives between February 16th, 2023 and April 30th, 2023 (Fig. 1).

### Campaign sites

Vaccination sites were selected from among urban and rural communities in six districts of the Boeny region: Ambatoboeny, Mahajanga I, Mahajanga II, Marovoay, Mitsinjo, and Soalala. Three of these districts (Marovoay, Mahajanga II, and Ambato-Boeny) were among the least covered by the national COVID-19 vaccination program at the time of the intervention, according to the national database. In Mahajanga I, we operated at the Public Health Regional Directorate (*Direction régionale de santé publique*, DRSP) where a dedicated site was set up as mass vaccination center, a so called “vaccinodrome”, and in three primary health care centers level 2 (CSB2: from the French *centres de santé de base niveau* 2), which offer essential obstetric care in addition to primary health care. Specifically, the CSB2s of Mahabibo, Mahavoky Sud, and Tanambao Sotema. A regular vaccination site was also created at the University Clinic Hospital (*Centre Hospitalier Universitaire*, CHU) Pzaga, in Androva, Mahajanga I. In the district of Marovoay, three CSB2s were selected based on previous community-based collaborations established by the team since 2020. Specifically, the urban CSB2 in Marovoay, and the rural CSB2s of Ankazomborona and Antanambao Andranolava. Two CSB2s in Mahajanga II (Belobaka and Boanamary) and two in Ambatoboeny (Andranofasika and Tsaramandroso) were selected because of their more populated catchment area and low vaccination coverage (national database).

### Continuous process evaluation

Informed by the principles of the DSF, we continuously monitored, discussed, and adapted the campaign’s implementation process by means of 1) VDC needs assessments, 2) weekly multi-sectoral stakeholder meetings, 3) key informant interviews e.g., with community health workers (CHWs), 4) problem-solving meetings of core staff with stakeholders, 5) monitoring of outcome indicators through weekly reports and meetings, considering the particularities of the implementation settings.

We developed a continuous assessment model of the VDC based on four evaluation measures: sourcing, storage, distribution, and administration. A communication plan consisting of weekly meetings with local authorities was established, in order to provide timely updates and exchanges on the progress of the COVID-19 vaccination roll-out. Continuous engagement of local authorities, CHWs and stakeholders generated a virtuous cycle of transparent feedback, discussion of progress, and adaptation of procedures.

### Outcome evaluation

We adopted different performance indicators to evaluate the outcomes of the CoBoGo vaccination campaign. Specifically, the number of outreach interventions, the number of administrated doses (primary and boosters) by week and month, distribution of doses by the geographic location, sex, and age group. In addition, we evaluated levels of awareness about COVID-19 vaccination, vaccine hesitancy, main sources of information, and perceptions of the amount of information received about COVID-19 vaccines. Further, implementation indicators, such as media coverage and number of public awareness activities were considered. Finally, sustainability indicators, such as staff trained, facilities equipped, the establishment and use of Standard Operating Procedures (SOPs) and awareness raising material were integrated into the Expanded Program on Immunization (EPI) program were also monitored and evaluated.

### Data collection

Data collection took place at different stages of the campaign with the aim of assessing both process and outcome indicators to inform, adapt, and to evaluate the campaign strategy within the context of the ecological system of Madagascar. Quantitative and qualitative data were collected during a pre-campaign phase, prospectively during the campaign Phase 1 and Phase 2, and at the end of the campaign.

### Qualitative data collection

Given the need for timely, real-time feedback on the implementation process and to inform stakeholders, qualitative data were collected throughout the campaign using a collaborative rapid assessment approach [17]. Qualitative data were generated during the individual interviews with key informants, such as with CHWs, and the discussions during the multi-sectoral stakeholder meetings. Embracing the concept of a participatory community-based approach, the latter included management staff and service providers of the health system, policy makers, community members and leaders, to seek feedback and discuss problems and possible solutions. Both the individual interviews with key informants as well as the stakeholder meetings were guided by a list of open-ended questions on informed by a priori topics of interest, such as vaccine availability, access to health facilities, medical requirements for vaccine delivery, communication strategies. The type of topics and questions varied as the campaign progressed. Prior to and at the beginning of the campaign the main focus was on questions, such as “How can we distribute available vaccine efficiently? What are the challenges?”, while as the issue of vaccination acceptance gained in importance, questions would revolve more around “How can we raise awareness around vaccinations? How can we encourage vaccine uptake? What are barriers? What are facilitators?”. Towards the end of the campaign one of the main topics concerned sustainability, and questions, such as “How can established capacities/strategies be integrated into future campaigns?”. The key informant interviews and the stakeholder meetings were conducted in French or Malagasy by at least a BNITM team representative and a Malagasy facilitator (e.g., supervisors or project coordinators). A total of 40 individual meetings and 36 stakeholder meetings were conducted. In the preparation phase of the campaign, meetings took place twice per week with the regional authorities, once the campaign started weekly between implementers and scientific team and for specific occasions (i.e. creation of new awareness raising material) with DRSP members, Health Promotion department staff, and CSB and hospital medical staff. Notes were shared across all participants and validated before final archiving. The individual interviews and discussions during the stakeholder meetings were not audio-recorded but detailed notes using field journals and meeting minutes were kept by the BNITM representative and the Malagasy facilitator.

### Quantitative data collection

Quantitative data collection involved two population-based cross-sectional surveys among probabilistic samples of the adult population (> 18 years old) living in Boeny, Madagascar. Data collection took place in September 2022 for the baseline survey and in May 2023 for the follow-up survey, and a total of 854 and 1034 participants were recruited, respectively.

Data were collected through face-to-face interviews using paper-based questionnaires and conducted by a team of 24 interviewers. The interviews were held in French or Malagasy, according to the participants’ preference. The variables collected included sociodemographic factors (sex, age group, urbanization (rural/urban), level of education (never attended school or incomplete primary education, primary or secondary education, secondary or university education), professional status (employed, not employed), perception of the financial situation during the COVID-19 pandemic (worsened, stayed the same), perception of the risk and severity of the COVID-19 disease and susceptibility to infection. Participants were asked to indicate the most reliable source(s) of information about COVID-19 vaccines and how they considered the amount of information received about COVID-19 vaccines (insufficient, sufficient, too much).

Survey participants who reported having heard or seen news about the COVID-19 vaccine before the survey were considered aware. Unvaccinated participants were considered hesitant if they answered “Definitely no” or “Possibly no” to the question: “If you had access to a vaccine against COVID-19, would you like to get vaccinated?”.

The fieldwork process was closely monitored by a team of three supervisors, to identify and correct the deviations from the established SOPs and ensure data quality in a timely manner. Survey databases were developed using the Kobotoolbox software [18].

Data on geographical and demographic distribution of COVID-19 vaccines were collected prospectively on a daily basis with the support of the DRSP Boeny, by accessing national-level data via the DHIS2 software platform [19].

### Data analysis

Qualitative data on the campaign process were analysed using a rapid deductive approach relying on notes and meeting minutes rather than transcripts (e.g. [20, 21]). To this end, to streamline the analysis process only one analyst reviewed and structured the notes and meeting minute. Informed by the a priori areas of interest, such as relating to the places of vaccine delivery, communication and awareness-raising strategies, rumours contributing to vaccine hesitancy, and acceptability, the analyst used a basic thematic approach to identify main issues across all individual interviews and discussions during the stakeholder meetings and structured these using a table.

To strengthen validity, key issues raised during the individual interviews would be presented by the BNITM interviewer(s)/facilitators/analyst(s) for discussion during the stakeholder meetings, or conversely, issues raised during the stakeholder meetings would be followed up on during the individual interviews with key informants. Finally, these key issues would be taken up and discussed during the problem-solving meetings with the BNITM core team. All findings were continuously shared with all stakeholders who had taken part in the process.

In order to assess campaign outcomes quantitative data analysis was performed using the software R version 4.3.1 [22]. Given the categorical nature of variables collected in population-based surveys, relative and absolute frequencies were used to summarize participants’ characteristics. The proportion of the population aware of COVID-19 vaccination and the level of vaccine hesitancy were estimated for each survey wave, in addition, main sources of information about vaccines used by the population and perception on amount of information received about COVID-19 were described. To estimate absolute change ($\Delta$) in outcome indicators over time, adjusting for sociodemographic characteristics of survey participants, we used generalized linear models of the Gaussian family with identity link function and robust standard errors [23]. To identify factors associated with vaccine hesitancy at baseline adjusted prevalence ratios (aPR) with 95% confidence intervals (CI 95%) were estimated using Poisson regression with robust standard errors [24].

To estimate the impact of the advanced strategy implemented during Phase 2 of the CoBoGo vaccination campaign (week 16) we developed an interrupted time-series analysis [25], fitting a negative binomial regression model with a weekly number of administrated vaccine doses as an outcome. Three independent variables were considered: a) time in weeks elapsed since the start of the CoBoGo campaign, to assess underlying temporal trends in Phase 1, b) binary variable indicating implementation of key adaptations (coded as 0 at Phase 1 and as 1 at Phase 2), to assess the immediate effect of the intervention (change in level), and c) time in weeks elapsed since implementation of key adaptations, to assess a change in the slope following key implementation adaptations in Phase 2 (ongoing effect). Stationarity and autocorrelation were examined using the autocorrelation function plot and the Box-Ljung test (Table S3, Figure S1, Additional file 1). There was no evidence of autocorrelation or non-stationarity. Estimates were presented in the form of rate ratios (RR), obtained by exponentiating model’s coefficients. In addition, to quantify the impact of the adaptive strategy, an expected number of doses administrated during Phase 2 in absence of key adaptations was estimated. Results were considered statistically significant at 5%.

## Results

Using the DSF as a heuristic device, in what follows we firstly describe the findings from our pre-campaign phase’s assessment, which helped in characterising Madagascar’s ecological context and practice setting in relation to the intervention. We then describe the initial strategy (Phase 1) in light of the findings of the earlier assessments. This is followed by a summary of the findings from the process evaluation, which prompted key adaptations of the initial strategy (Phase 2). Finally, we present the results from our outcome evaluation (Fig. 1). The interventions operated at each site, in terms of logistics and infrastructure, workforce, community engagement, and stakeholder collaboration, all of which are summarised in Table 1.

**Table 1:** Summary of interventions operated at each vaccination site

	**Mahajanga I**					**Marovoay**			**Mahajanga II**		**Ambato-Boeny**	
	**Vaccinodrome**	**CHU Pzaga (Androva)**	**Mahavoky Sud**	**Tanambao Sotema**	**Tsararano Ambony**	**Marovoay-centre**	**Ankazoborona**	**Antanamboa**	**Belobaka**	**Boanamary**	**Andranofasika**	**Tsaramandroso**
**Logistic & Infrastructures**		☑							☑	☑	☑	☑
**Workforce**						☑	☑	☑	☑	☑	☑	☑
**Community engagement**	☑	☑	☑	☑	☑	☑	☑	☑	☑	☑	☑	☑
**Stakeholder Collaboration**	☑	☑	☑	☑	☑	☑	☑	☑	☑	☑	☑	☑

### Pre-campaign phase

#### Characteristics of the ecological system and practice setting

Our territorial assessment allowed to identify two main scenarios in terms of available infrastructures: (i) CSBs appropriately resourced to perform a vaccination campaign, (ii) functional health structures for storage of vaccines and waste disposal, (iii) CSBs that required infrastructural intervention to allow the conduct of the campaign. The main elements identified as barrier for the campaign were related to a stable electricity supply that would impact directly both storage and delivery of the vaccines. Among the infrastructures evaluated, we found no barriers for the implementation of the campaign in the CSBs of the districts of Mahajanga I and II, Mitsinjo, and Soalala. The vaccinodrome at the DRSP in Mahajanga I and the CHU Pzaga were selected as central facilities to store vaccines and to operate the waste disposal respectively. For waste disposal, minor interventions were needed to be put in place, e.g., while the CHU Pzaga was already sufficiently equipped the facility had not been operational. Finally, the CSBs of the districts of Marovoay and Ambato-Boeny required infrastructural interventions in order to guarantee adequate vaccine delivery. Some of the areas for improvement identified in terms of supply management, included inefficiencies in tracking inventory lists, maintaining equipment, and overseeing management practices. Findings from stakeholder meetings, KII and discussions with community members collectively shed light on various ecological aspects related to both direct elements of vaccine administration, such as insufficient medical devices, staffing shortages, inadequate quality control measures and, lack of user-friendly data collection tools at vaccination hubs, as well as those more related to the surroundings such as the possibility to reach remote locations, community beliefs, and attitudes towards healthcare. Insights gleaned from discussions with policymakers underscored critical ecological considerations, highlighting the imperative to enhance sanitary waste disposal and bolster pharmacovigilance preparedness.

Through a community-based survey, we assessed COVID-19 vaccine awareness as well as common sources of information, and attitudes towards vaccination. A total of 854 participants aged 18 years or more were recruited in the first wave of the COVID-19 vaccine awareness survey, 48.1% male and 51.9% female. Participants’ characteristics are summarized in Table S1 (Additional file 1).

**Fig. 2:**
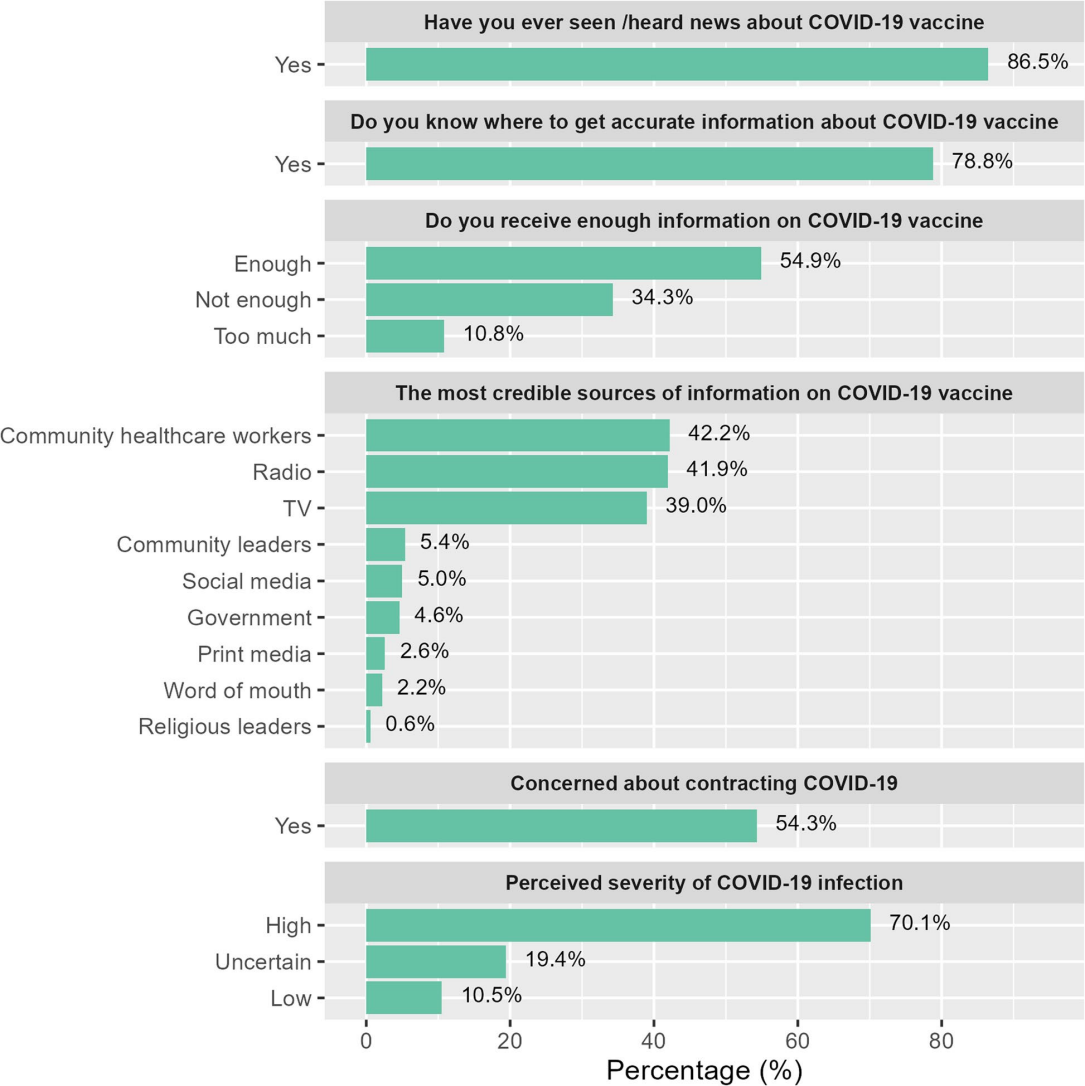
Population awareness, vaccine hesitancy, and perceptions regarding COVID-19 prior to the CoBoGo campaign

Prior to CoBoGo, the population level of awareness about COVID-19 in the Boeny region had been high, with 86.5% of survey respondents reporting to have heard or seen news about COVID-19 vaccines, and 78.8% stating that they know where to get accurate information about COVID-19 vaccines (Fig. 2). The survey identified CHWs (42.2%), Radio (41.9%), and TV (39.0%) as the most frequently used trusted sources of information about vaccines among the population. Only 5.0% of participants considered social media as a reliable source of information on COVID-19 vaccination. More than half of the respondents (54.9%) reported to have received enough information about COVID-19, while 34.3% perceived the amount of information received as not enough, while 10.8% stated to have received too much information.

Among the unvaccinated participants, 54.3% of the respondents expressed concerns about contracting COVID − 19, and 70.1% considered COVID − 19 to be a severe disease. However, 50.4% of the respondents reported to be hesitant towards COVID-19 vaccinations. Levels of vaccine hesitancy varied among population subgroups. Female sex (aPR = 1.31, CI 95%:1.13; 1.56), higher education (aPR = 1.29, CI 95%: 1.01; 1.64), lack of trust in health authorities (aPR = 1.52, CI 95%: 1.30; 1.78), low perceived risk of contracting COVID-19 (aPR = 1.50, CI 95%: 1.24; 1.80), perceived low severity of the COVID-19 disease (aPR = 1.41, CI 95%: 1.15; 1.73) were associated with higher vaccine hesitancy (Table S2, Additional file 1).

Discussions with community members gave further insights into potential barriers of vaccine acceptance within the communities, such as the influence of the infodemic, and rumours about the efficacy of the vaccine, and adverse effects. Additionally, they allowed us to identify suitable modalities for vaccination delivery, such as through mobile units at marketplaces.

### Phase 1: initial strategy – key adaptations to the standard approach

Based on the elements that were identified by both the qualitative and quantitative pre-campaign assessment data, an initial strategy was designed to start the vaccination. Key adaptations to the standard approach implemented prior to the campaign were organised into three main pillars, addressing respectively (i) logistics and supply, (ii) awareness raising initiatives, and (iii) re-organization of medical staff.

The first pillar covered initially those CSBs, that required infrastructure enhancement. These were capacitated through the installation of solar generators to mitigate energy instability. Additionally, a standardized supply management system was implemented, including the introduction of a material inventory, and a quality management system, incorporating user-friendly tools, such as site-specific material lists and weekly consumption tables, which seamlessly integrated into the routine of the CSBs.

As part of the second pillar, an awareness raising initiative was implemented. A total of ten radio spots were designed by 14 community ambassadors, selected from among youth champions engaged within local activist groups, such as students, scouts, religious, and women’s associations. The DRSP Health Promotion Department revised and approved the messages that were delivered through three radio stations five times per day for a total of 20 weeks.

The ambassadors played an essential role in our participatory approach, which formed the basis of our campaign. After an exchange session, including definition of needs by the community and targeted trainings to address the communication with the community, the ambassadors promoted the vaccination campaign through community-based initiatives, such as university football matches, festivals, and market days. They additionally coordinated their work with CHWs in mobilizing community members to produce a snowball effect of the campaign by which every member of the community was simultaneously recipient and deliverer of the campaign through community meetings and door-to-door visits.

Finally, the third pillar involved an adapted team organization for the delivery of the vaccines in order to optimize the use of human resources both in terms of roles, specific expertise, and knowledge of the territory. Specifically, three doctors were assigned coordinating roles based on their expertise and areas of focus. One doctor oversaw vaccination activities, collaborating closely with the DRSP and CSB2 chiefs to ensure adherence to SOPs and alignment with the DRSP’s EPI. Another doctor coordinated rural sites, overseeing awareness initiatives, and vaccination activities whilst conducting regular monitoring visits to ensure well-equipped facilities and effective waste management. A third doctor led the awareness team, comprised of Malagasy ambassadors and CHWs, who facilitated communication and collaboration among team members to maximize the impact of the awareness campaigns.

#### Campaign process evaluation

Informed by the principles of the DSF, we implemented a comprehensive process evaluation that unfolded throughout the entirety of the campaign. This evaluation involved continuous monitoring, discussion, and adaptation of the campaign’s implementation process. Weekly multi-sectoral stakeholder meetings facilitated collaboration between CSBs chiefs, and healthcare providers fostering coordinated efforts to address challenges such as vaccine hesitancy and misinformation. During the Pre-campaign Phase and between Phase 1 and 2, VDC needs assessments revealed critical gaps in vaccine distribution infrastructure, emphasizing the urgent need for improved transportation and additional medical devices, such as vaccine carriers to effectively reach remote areas. As a result of these assessments, we recognized the necessity to adapt our strategy. Problem-solving meetings involving core staff and stakeholders led to innovative solutions for logistical challenges, such as a community-led awareness campaigns and the introduction of mobile vaccination clinics in Phase 1, and their expansion, in terms of number of initiatives, during Phase 2. The ongoing monitoring of outcome indicators allowed for real-time assessments of the campaign’s progress, enabling us to identify areas where strategy adjustments were necessary to optimize vaccine coverage and address emerging issues throughout the project’s life cycle.

### Phase 2: key adaptations

Informed by the comprehensive process evaluation conducted throughout the campaign, we not only made several key adaptations during the initial phase of the campaign, but also within each implementation pillar as we transitioned into Phase 2.

#### Logistics and infrastructure

Recognizing logistical hurdles in vaccine transportation and infrastructural needs, we increased frequency of vaccine distribution activities from biweekly in Phase 1 to daily in Phase 2. This involved extending transportation to ensure access to remote areas and providing additional vaccine carriers. In Phase 2, the advanced strategy was extended to include four additional CSBs in Mahajanga II (Belobaka and Boanamary,) and two in Ambato-Boeny (Andranofasika and Tsaramandroso).

#### Community engagement

Continuation of community-led awareness campaigns in Phase 2, due to their successful implementation in Phase 1.

#### Stakeholder collaboration

Leveraging the success of weekly multi-sectoral stakeholder meetings in fostering collaboration between CSB chefs and healthcare providers, these too were continued without significant changes in Phase 2.

#### Health workforce strengthening

Responding to the need for increased human resources in the rural sites, we deployed a higher number of CHWs for awareness-raising activities during Phase 2.

By aligning our adaptations with the pillars identified through our continuous process evaluation, we ensured a responsive approach in addressing challenges as they arose, and to optimize vaccine coverage.

### Campaign indicators: outcome evaluation

#### Distribution of vaccine doses

**Fig. 3:**
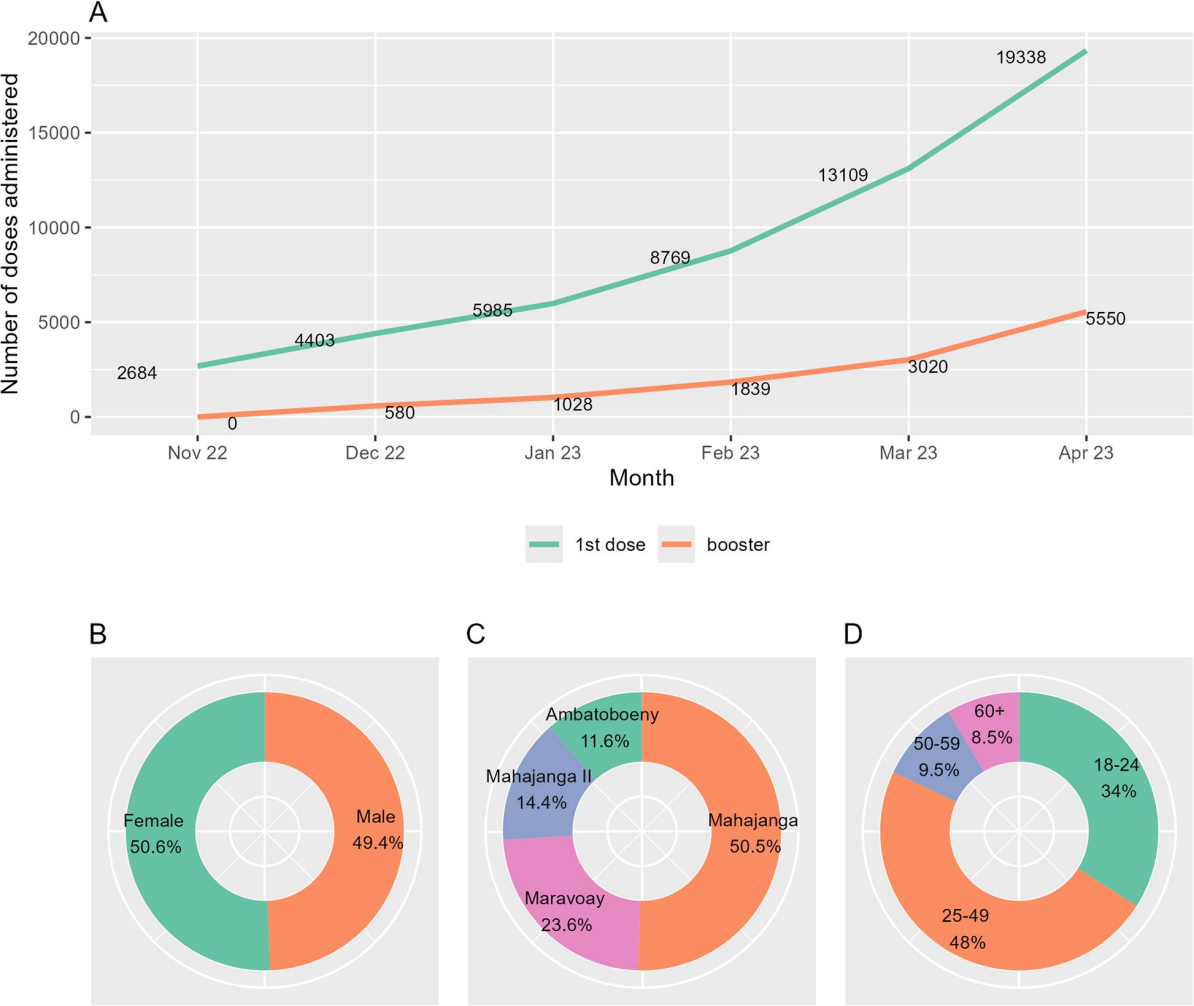
Distribution of COVID-19 vaccine doses deployed during the CoBoGo campaign. Legend: **A** Cumulative monthly number of 1st doses and booster vaccine doses, **B** Distribution of doses by sex, **B** Distribution of doses by region, **B** Distribution of doses by age group

During the CoBoGo campaign, a total of 566 outreach activities were conducted, and a total of 24,888 COVID-19 vaccine doses administered, 19,338 first doses and 5550 boosters, ranging from 2684 to 8759 doses per month (Fig. 3, panel A). The distribution of doses by sex was balanced, 49.4% for male vs 50.6% for female (Fig. 3, panel B). Of all the vaccine doses, 50.5% were administrated in Mahajanga, 23.6% in Marovoay, 14.4% in Mahajanga II, and 11.6% in Ambato-Boeny (Fig. 3, panel C). The CoBoGo campaign mostly reached middle-aged and young individuals, the majority of the vaccinated were 25–49 years old (48%) and 18–24 years old (34%), while only 9.5% and 8.5% of doses were distributed among 50–59 and 60 + years old, respectively (Fig. 3, panel D).

#### Comparison between Phase 1 and Phase 2

**Fig. 4:**
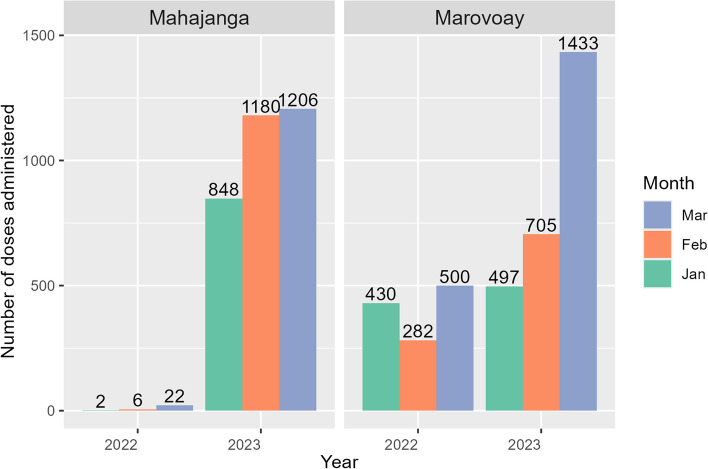
Comparison of CoBoGo campaign (2023) vs. standard approach (2022) in first trimester vaccine deliveries

Comparing the performance of the CoBoGo campaign in first trimester of 2023 to a previous standard approach implemented in the first trimester of 2022, we observed a considerable increase in the number of administered doses in the CSB2s of Mahajanga I (Tanambao Sotema, Mahavoky Sud and Tsararano Ambony) and more than a 2.2-fold increase in Marovoay (Morafeno, Antanambao and Ankazomborona) (Fig. 4).

**Fig. 5:**
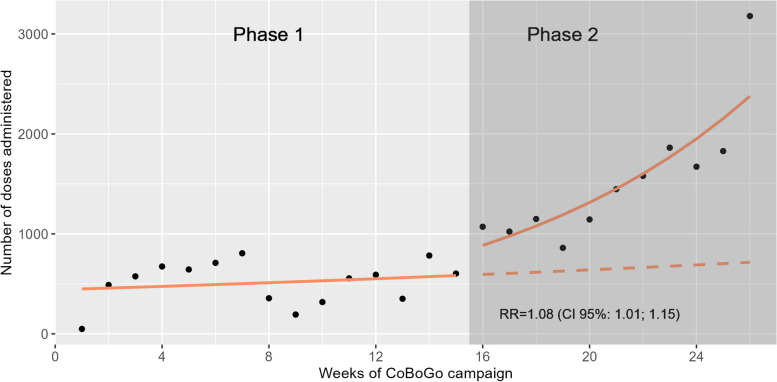
Weekly COVID-19 vaccine deployment trends: CoBoGo Phases 1 and 2

Comparing the performance of the CoBoGo campaign following the implementation of Phase 2 vs. Phase 1 we observed a statistically significant change in trend, with an 8% increase in the number of weekly administered doses (RR = 1.08, CI 95%: 1.01; 1.15) (Fig. 5). Baseline trend (RR = 1.02, CI 95%: 0.96; 1.08) and change in level (RR = 1.38, CI 95%: 0.89; 2.12) were not statistically significant (Table S3 Additional file 1). We estimated that under the hypothetical scenario without implementation of the adapted strategy of Phase 2 between weeks 16 and 26, only 7184 vaccine doses would have been delivered to the population, while with our adaptation we were able to deliver 16,815 COVID-19 vaccines.

### Awareness campaign

#### Variability of vaccine awareness and hesitancy outcomes

**Fig. 6:**
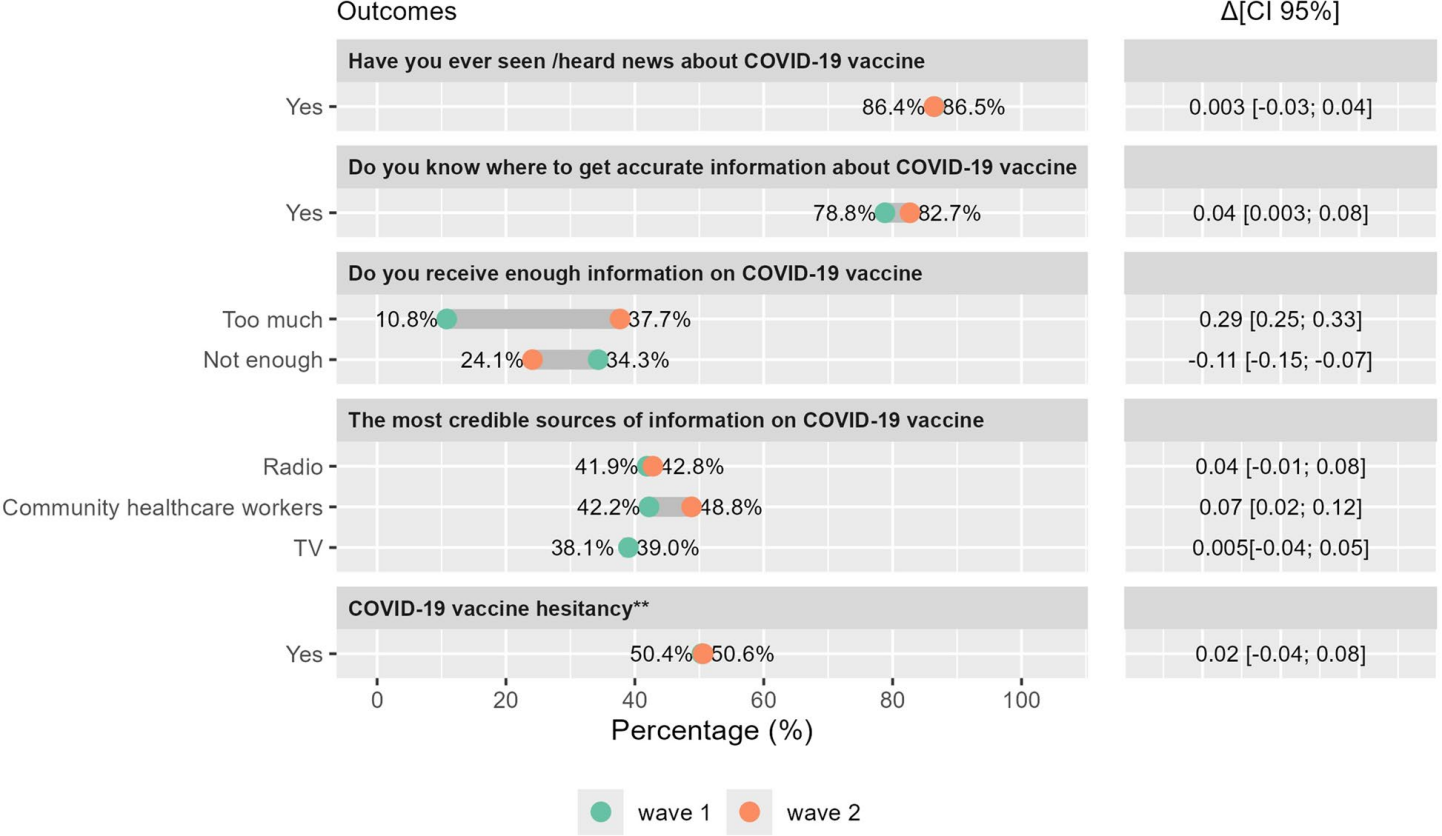
Change in outcomes ($\Delta [\text{CI }95{\%}])$ following CoBoGo vaccination campaign implementation. Legend: **Model adjusted for sex, age group, urbanization, education, occupation, perceived financial situation during the COVID-19 pandemic, living with children under 5, concerns about contracting COVID-19, perceived severity of COVID-19 infection, and trust in health authorities

A total of 1034 participants aged 18 years and older were recruited into the second COVID-19 awareness survey wave. Participants’ characteristics are summarized in Table S1 (Additional file 1). No significant changes in COVID-19 vaccine awareness were observed at population level after the CoBoGo campaign. The proportion of awareness was 86.4% in the second survey wave, compared to 86.5% in the first wave ($\Delta$=0.003, CI 95%: −0.03; 0.04) (Fig. 6). For the unvaccinated population, the level of vaccine hesitancy remained high, following CoBoGo, 50.6% of unvaccinated respondents reported to be hesitant to get vaccinated against COVID-19 in the second survey wave, compared to 50.4% estimated at baseline, ($\Delta$=0.02, CI 95%: −0.04; 0.08). In contrast, the proportion of those who knew where to get accurate information on COVID-19 vaccination increased after CoBoGo, from 78.8% to 82.7% ($\Delta$=0.04, CI 95%: 0.003; 0.08).

Radio (42.8%), TV (38.1%) and CHWs (48.8%) remained the most frequently mentioned sources of information on COVID-19 vaccines, however, the proportion of the population recognizing CHWs as the most reliable source of information increased (△=0.07, CI 95%: 0.02; 0.12). Population perceptions regarding the amount of available information also changed over time: the proportion of those not receiving enough information on COVID-19 decreased from 34.3% to 24.1%, (△=-0.11, CI 95%: -0.15; -0.07), whereas the proportion of those who reported to have received too much information increased from 10.8% to 37.7% (△=0.29, CI 95%: 0.25; 0.33) (Fig. 6).

### Sustainability outcomes

#### Staff trained

A total of 340 health care staff were trained in topics and research methodologies related to vaccines and vaccination. A total of 260 CHWs were trained on awareness-raising strategies. The 42 health care workers permanently employed by the regional vaccination program were trained in vaccine administration and management of adverse events following immunization as well as in safety procedures and quality management of the stock. Twenty-four interviewers and six researchers were trained in research methods and primary qualitative and quantitative data. Finally, a total of 14 young ambassadors selected to engage communities through peer communication, were trained in addressing communication issues to mitigate rumours and the infodemic. All of the CHWs and vaccination program team, such as vaccine administrators and nurses, were permanently employed by the MoH. All other staff members were selected from a pool of non-permanent staff within the region as identified by local stakeholders. One advantage of training both MoH and local staff is that both are available for future implementation initiatives and operational research projects.

#### Facilities equipped

A total of 12 governmental facilities were equipped and refurbished. Of those, 10 were at primary level of care (CSBs). The specific interventions operated included the reinforcement of the power and cold chain through the installation of solar panels and −80°C freezers, the structure of the waste management at both decentralised and central level so as the introduction of tools for the weekly supply monitoring to mitigate stockouts. Additionally, equipment for mobile vaccination hubs, designed to be sustainable and versatile, was supplied for placement in CSBs and outreach activities. All materials were sourced from local manufacturers and allocated to the DRSP’s EPI at the project’s conclusion to sustain ongoing activities.

#### SOPs and awareness raising material

A total of four SOPs were established in the frame of the campaign in collaboration with the Boeny DRSP’s EPI. These addressed: 1. vaccination procedures, 2. adverse event management, 3. pharmacovigilance, and 4. the implementation of sanitary waste disposal. They have been formally integrated within the vaccination plan of the Boeny DRSP’s EPI.

In addition, a collection of information material, including visual and audio messages, were archived within the Health Promotion Department of the DRSP of Boeny upon direct validation by the MoH. A clear workflow was created to build and validate awareness-raising content, which can be sustainably reused for future initiatives.

## Discussion

This study provides details on the strategic approach and steps that were undertaken during the implementation of a COVID-19 vaccination campaign in the rural region of Boeny in Madagascar, with the aim of providing guidance for future mass vaccination campaigns in this and similar settings. To the best of our knowledge, this is the first study reporting on an evaluation of a COVID-19 vaccination campaign in a low- and middle-income country based on the concept of the DSF.

Firstly, it shows how the principles of the DSF were incorporated and put into practice. The underlying rationale of the DSF is that adapting an intervention to better fit the context, as opposed to maintaining fidelity to the original programme, may enhance outcomes [26]. In order to assess and adapt the intervention, both process and outcome indicators were being monitored and evaluated. To this end, our campaign used an innovative approach by combining epidemiological and social research methods (i.e., community-based surveys, qualitative key informant interviews) with an implementation science theoretical framework (i.e., DSF) to collect and analyse data on a continuous basis. Based on these findings, key adaptations were made to the original campaign strategy in response to contextual changes, including communication and outreach strategies that proved to impact on major campaign outcomes. In fact, if our data show that this type of initiatives do not manage to produce societal changes at a large scale, they are capable of producing improvements in campaign outcomes, such as vaccination coverage.

Secondly, in alignment with a dynamic understanding of sustainability, rather than focusing sustainability efforts solely on maintaining compliance with specific interventions (in this case COVID-19 vaccinations), we focused our sustainability efforts on having in mind future shifts in use and type of interventions to improve public health outcomes (as opposed to campaign outcomes only). Training staff in vaccine administration, operational research, monitoring, equipment maintenance, and community engagement ensures continuity and readiness for future vaccine or public health campaigns. For instance, since COVID-19 vaccination programs focus on adults, who are typically not the priority target group for vaccination in SSA, our campaign strategy provides valuable insights for future adult vaccination programs. One pressing example of these, includes HPV vaccinations in LMICs [27, 28], which are largely behind schedule as set out by the WHO targets [29].

Finally, multi-sectoral stakeholder engagement and fostering local ownership was crucial for the success of the campaign and setting the path for the future. Based on our experience we argue that the risk of smaller scale non-governmental organization (NGO) driven initiatives not being able to sustain the impact of their interventions [30], can be reduced by coordinating these with local stakeholders, such as authorities, policy makers, communities, and direct beneficiaries from the start. Indeed, the approach of this vaccination campaign draws on the authors’ experience and lessons learnt during previous work with international NGOs such as *Medecins Sans Frontiers* [31], their collaborative implementation networks established in the region, and the operational research expertise built together within the frame of the Madagascar-BNITM partnership over time. This innovative approach shows that not only a multidisciplinary approach but also a multi-sectoral one is critical for the successful implementation of health programs. This study represents a unique opportunity to help implementers in the field of vaccinology and health programs to conceptualize interventions to maximise outputs in similar contexts, such as rural areas of SSA countries as well as in more remote communities of the global north that have experienced similar constraints during the implementation of COVID-19 vaccination initiatives [32, 33].

Despite its strengths, our study is not without limitations. Firstly, we cannot exclude a social desirability bias associated with self-reported survey data. Additionally, the quantitative data are drawn from repeated cross-sectional surveys, meaning that conclusions cannot be made regarding the causality of relationships. Given time constraints and the need for real-time qualitative data to continuously inform our implementation process and stakeholders, a rapid qualitative assessment approach was used, which may have affected the balance between efficiency and rigor, for example, the textual data were only reviewed by one analyst, however, findings were discussed by the larger team and at stakeholder meetings. Finally, comparisons with other studies and other contexts should be made with caution, due to the unique conditions related to specific contexts that might limit the generalisability of the findings.

## Conclusion

Our initiative provides practical guidance on how to successfully implement collaborative, multi-sectoral vaccination campaigns, which integrate health care services with a proactive outreach and community-driven campaign. It demonstrates how a theory-based adaptive implementation approach can enhance vaccination coverage for COVID-19 vaccinations in Madagascar, even if the impact on vaccine hesitancy remains limited. While our strategy has particularly important implications for other settings with limited resources, it can also be relevant for improving vaccination coverage in areas where resources are not necessarily the bottleneck. Finally, this study provides valuable directions on how to improve the global concept of vaccination programs for adults more broadly.

## Supplementary Information

Supplementary Material 1.

## Data Availability

Raw data used in the current study are available from the corresponding author on reasonable request and will be freely available to researchers who wish to use them for non-commercial purposes, without breaching the confidentiality of participants. The code used in the current study are available from the corresponding author on reasonable request and will be freely available to researchers who wish to use them for non-commercial purposes, without breaching the confidentiality of participants.

## Abbreviations

aPR: Adjusted prevalence ratio
BNITM: Bernhard Nocht Institute for Tropical Medicine
CERBM: National Biomedical Research Ethics Committee in Madagascar
CHU: Centre Hospitalier Universitaire
CHW: Community Healthcare Worker
CI 95%: 95% Confidence interval
CoBoGo: COVID-19 vaccination campaign in the Boeny region of Madagascar: paving the road for worldwide vaccination coverage goal
COVAX: COVID-19 Vaccines Global Access
COVID-19: COronaVIrus Disease 19
CSB: Centre *de* Santé de Base
DHIS2: District Health Information System 2
DRSP: Direction régionale de santé publique
DSF: Dynamic sustainability framework
EPI: Expanded Program on Immunization
IT: Information Technology
KII: Key informant interview
LMIC: Low- and middle-income countries
MoH: Ministry of Health
NGO: Non-governmental organization
RR: Rate ratios
SOP: Standard operating procedures
SSA: Sub-Saharan Africa
VC: Vaccination campaign
VDC: Vaccine distribution chain
WHO: World Health Organization
