# Supplementary material for: Optimizing vaccine uptake in sub-Saharan Africa: a collaborative COVID-19 vaccination campaign in Madagascar using an adaptive approach
Source: Implement Sci. 2025 Jan 9;20:2. doi: 10.1186/s13012-024-01412-5 (PMC11720884; doi:10.1186/s13012-024-01412-5)
Supplement: Supplementary file 1 — Supplementary Material 1. [file 13012_2024_1412_MOESM1_ESM.docx]

Table S1. Participants' characteristics by survey wave

| **Characteristics** | **Wave 1**  **(N=854)** | **Wave2**  **(N=1034)** |
| --- | --- | --- |
| **Sex** |  |  |
| Female | 443 (51.9%) | 557 (53.9%) |
| Male | 411 (48.1%) | 477 (46.1%) |
| **Age group** |  |  |
| 18-29 | 287 (33.6%) | 458 (44.3%) |
| 30-39 | 218 (25.5%) | 240 (23.2%) |
| 40+ | 349 (40.9%) | 336 (32.5%) |
| **Urbanization** |  |  |
| Rural area | 417 (48.8%) | 502 (48.5%) |
| Urban area | 437 (51.2%) | 532 (51.5%) |
| **Living with children under 5 years old** |  |  |
| No | 556 (65.1%) | 694 (67.1%) |
| Yes | 296 (34.7%) | 340 (32.9%) |
| **Education** |  |  |
| No education/incomplete primary school | 178 (20.8%) | 216 (20.9%) |
| Primary/middle school | 325 (38.1%) | 356 (34.4%) |
| Secondary school/university | 351 (41.1%) | 461 (44.6%) |
| **Perceived financial situation during the COVID-19 pandemic** |  |  |
| Not worse | 210 (25.5%) | 491 (51.6%) |
| Worse | 614 (74.5%) | 460 (48.4%) |

Table S2. Prevalence of COVID-19 vaccine hesitancy and associated factors prior of the CoBoGo VC

|  | **% hesitancy**  **(CI 95%)** | **Crude** | | **Adjusted** | |
| --- | --- | --- | --- | --- | --- |
|  |  | **PR** | **CI 95%** | **aPR** | **CI 95%** |
| **Sex** |  |  |  |  |  |
| Female | 56.1(50.4;61.7) | 1.29 | (1.08, 1.53) | 1.31 | (1.11; 1.56) |
| Male | 43.6(37.4;49.9) | ref |  | ref |  |
| **Age group** |  |  |  |  |  |
| 18-29 | 54.9(47.9;61.9) | ref |  | ref |  |
| 30-39 | 48.7(40.8;56.6) | 0.89 | (0.72; 1.09) | 0.90 | (0.73; 1.10) |
| 40+ | 47.4(40.3;54.5) | 0.86 | (0.71; 1.05) | 0.91 | (0.74; 1.12) |
| **Urbanization** |  |  |  |  |  |
| Rural area | 48.4(42.5;54.3) | ref |  | ref |  |
| Urban area | 52.7(46.6;58.8) | 1.09 | (0.92; 1.29) | 0.91 | (0.76; 1.09) |
| **Education** |  |  |  |  |  |
| No education/  incomplete primary school | 45.8(36.8;54.8) | ref |  | ref |  |
| Primary/middle school | 45.0(38.4;51.6) | 0.98 | (0.77; 1.26) | 1.06 | (0.83; 1.34) |
| Secondary school/university | 59.2(52.4;66) | 1.29 | (1.03; 1.62) | 1.29 | (1.01; 1.64) |
| **Perceived financial situation during the COVID-19 pandemic** |  |  |  |  |  |
| Not worse | 53.6(44.9;62.3) | ref |  | ref |  |
| Worse | 48.7(43.8;53.7) | 0.91 | (0.75; 1.1) | 0.87 | (0.72; 1.05) |
| **Living with children under 5 years old** |  |  |  |  |  |
| No | 50.4(45.2;55.7) | ref |  | ref |  |
| Yes | 50.8(43.7;57.9) | 1.01 | (0.85; 1.2) | 1.02 | (0.85; 1.23) |
| **Concerned about contracting COVID-19** |  |  |  |  |  |
| No | 64.2(58.2;70.2) | 1.67 | (1.41; 1.99) | 1.5 | (1.24; 1.8) |
| Yes | 38.3(32.7;44) | ref |  | ref |  |
| **Perceived severity of COVID-19 infection** |  |  |  |  |  |
| Low | 74.5(63;86.1) | 1.68 | (1.39; 2.04) | 1.41 | (1.15; 1.73) |
| Uncertain | 60.4(51.1;69.7) | 1.36 | (1.13; 1.65) | 1.18 | (0.97; 1.44) |
| High | 44.3(39.3;49.3) | ref |  | ref |  |
| **Trust in health authorities** |  |  |  |  |  |
| No/ uncertain | 69.2(61.7;76.8) | 1.58 | (1.35; 1.85) | 1.52 | (1.30; 1.78) |
| Yes | 43.8(38.9;48.7) | ref |  | ref |  |

Table S3. Negative binomial model for weekly number of administrated vaccine doses by the CoBoGo campaign

| Variable | RR | CI 95% |
| --- | --- | --- |
| Time in weeks since the start of the CoBoGo (baseline trend) | 1.02 | (0.96, 1.08) |
| Implementation of the key adaptations (change in level) | 1.38 | (0.89, 2.12) |
| Time in weeks since the implementation of the key adaptations (change in slope) | 1.08 | (1.01, 1.15) |

AIC= 383.81, Box-Ljung test p-value= 0.4053

Figure S1. Negative binomial model for weekly number of administrated vaccine doses by CoBoGo campaign: the autocorrelation function plot**
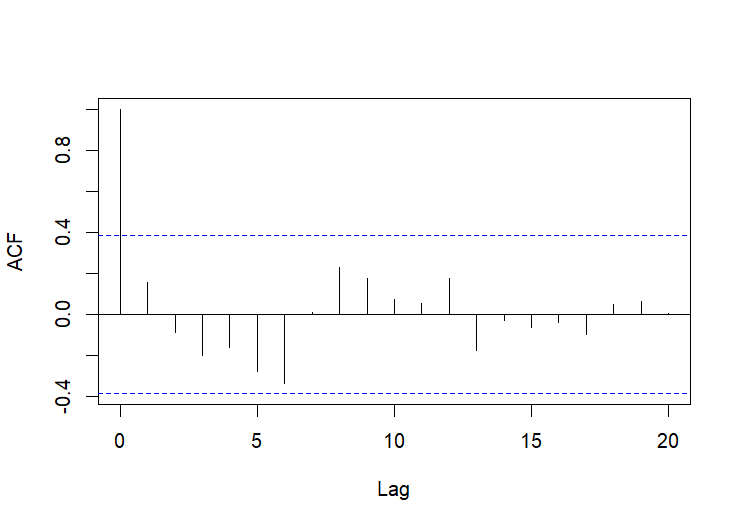
**
